# Factors Associated with Changes in Peripapillary Retinal Nerve Fibre Layer Thickness in Healthy Myopic Eyes

**DOI:** 10.1155/2021/3462004

**Published:** 2021-12-27

**Authors:** Jianli Du, Yang Du, Yanyan Xue, He Wang, Yaping Li

**Affiliations:** ^1^Department of Ophthalmology, The Second Hospital of Jilin University, Changchun, China; ^2^Department of Ophthalmology, The First Hospital of Xi'an, Northwest University, Xi'an, China; ^3^Department of Ophthalmology, The Third People's Hospital of Dalian, Dalian Medical University, Dalian, China

## Abstract

Myopic people face an elevated risk of primary open angle glaucoma. Changes in the fundus in people with high myopia often lead to misdiagnosis of glaucoma, as this condition has many clinical signs in common with myopia, making the diagnosis of glaucoma more challenging. Compared to reduction of the visual field, a decrease in retinal nerve fibre layer (RNFL) thickness occurs earlier in glaucoma, which is widely considered useful for distinguishing between these conditions. With the development of optical coherence tomography (OCT), RNFL thickness can be measured with good reproducibility. According to previous studies, this variable is not only affected by axial length but also related to the patient's age, gender, ethnicity, optic disc area, and retinal blood flow in myopia. Herein, we intend to summarize the factors relevant to the RNFL in myopia to reduce the false-positive rate of glaucoma diagnosis and facilitate early prevention of myopia.

## 1. Introduction

Myopia is one of the most common ocular diseases worldwide. The prevalence of myopia is estimated at approximately 20% to 30% among children in Singapore [1], and this figure is expected to increase in the future. Generally, high myopia is defined as occurring when the spherical equivalent reaches at least 6 dioptres (D) and the axial length elongates above 26 mm. The anatomic features of myopia that involve changes in the optic nerve, such as a large optic disc and optic tilt, also increase the risk of primary open angle glaucoma. Although measuring the visual field is the gold standard for the diagnosis of glaucoma, structural changes such as thinning of the RNFL, which consists mainly of ganglion cells, precede functional defects. Structural change is observed six years prior to any detectable visual field defects in up to 60% of eyes [2]. Additionally, the RNFL plays a role in the pathological mechanism of myopia. Therefore, it can be used as a tool to distinguish glaucoma from myopia, diagnose early glaucoma, and better understand the progression of myopia.

Optical coherence tomography (OCT) allows imaging of the RNFL with high resolution in a noninvasive and even contact-free manner. This technique was first introduced in 1991 [3]; since then, its development has progressed through several generations. The latest types of equipment, including swept-source OCT (SS-OCT) and OCT angiography (OCTA) apparatuses, are widely recognized in clinical practice. SS-OCT uses a longer wavelength (1050–1060 nm) than conventional OCT, allowing the beams to penetrate the retinal pigment epithelium (RPE) and image deeper tissues such as the choroid and posterior sclera [4, 5]; meanwhile, conventional OCT, with a shorter wavelength of approximately 840 nm, has difficulty measuring these tissues [6]. It is also suitable for the measurement of eyes with cataracts due to its reduced attenuation from scattering in opaque media [7]. OCTA captures three-dimensional images of the ocular vasculature by acquiring repeated B-scans from the same retinal location [8, 9] and detecting the movement of erythrocytes within blood vessels [10]. Depending on the intensity of the signal, OCT achieves various degrees of tissue penetration. Swept-source OCTA (SS-OCTA), which has a longer wavelength, can better measure the retina and choroid vasculature, while spectral-domain OCTA (SD-OCTA) may cause false-positive measurements of the deep vasculature because its wavelength is approximately 840 nm [11]. Accurate measurement of the RNFL helps investigators better understand the pathogenesis of myopia. However, the built-in normative databases of most OCT devices are suitable only for adults without high myopia and will cause false-positive results if applied to patients with high myopia. These measurement errors may hinder the diagnosis of glaucoma among myopic people. There are many studies examining various factors associated with RNFL in myopia. However, there has not been a review summarizing what these factors are and how they are associated with the RNFL. Therefore, examining factors associated with RNFL in myopia is necessary to build a new database that is suitable for myopic people, and many factors should be considered in RNFL measurements.

## 2. Age

When people receive OCT examination, examiners should record their age first. It not only helps us discern the target patient from those who have the same names but also compares them to the corresponding age-matched normal database to exclude age confounders.

A cross-sectional observational study of 82 Taiwanese adults over 65 years old found that the global RNFL thickness was reduced by 4.97 *μ*m every ten years [12]. Among different age groups, a 10-year gap could cause the average RNFL thickness to decrease from 1.5 to 2.5 *μ*m [13]. This difference between the above studies may be attributed to the latter subjects being younger. Histological data have also revealed nearly 4000–5000 optic nerve fibres lost per year [14]. However, this decline along with the increase in age did not occur equally around the optic disc. The decrease in superior and inferior sectors was more obvious in the position where the main retinal blood vessels resided, while the nasal and temporal halves did not show this relevance, suggesting a redistribution of axons with age. The mechanism by which these two areas are preferentially affected is unclear. Some investigators have attributed it to the more rapid atrophy of larger-diameter nerve fibres; these two quadrants are where large-diameter fibres are most abundant [15]. In addition, the quadrants containing the main retinal blood vessels tend to decline most with age [16]. The relatively rich blood supply prevents damage in highly myopic eyes. In myopic subjects aged 20 to 34 years, Singh et al. [17] did not find any association between age and RNFL thickness.

Sectoral RNFL thickness was not equally affected by age. Different age ranges, including adults and children, also showed significant differences. Among Chinese children, the mean RNFL thickness did not show any significant difference between 7-year-old children and 12-year-old ones, measuring 102.01 ± 8.02 *μ*m and 103.08 ± 9.01 *μ*m, respectively [18], and the degree of myopic shift between this age range did not show any change in RNFL thickness. This result indicated that the negative association between RNFL thickness and age may not start at this early age. RNFL thinning became more significant when participants reached 41 years old, and the impact of age on RNFL thickness did not show any significant difference in longer or shorter eyes. The superior quadrant was both the earliest sector affected (by the age of 35 years) and the sector with the highest rate of progression [19]. In contrast, the nasal and temporal quadrants did not show this same pattern of change with age [12]. The superior and inferior quadrants were found to lose thickness more quickly due to the weakness of the lamina cribrosa in these two areas [20]. The RNFL thickness in this quadrant increased by 0.308 *μ*m/yr [21], because nonneuronal components, such as glial cells, account for part of the RNFL.

Considering the way age affects the RNFL in myopia, it is crucial to enter the correct date of birth, which can prevent irregularities in RNFL thickness measurement that could lead to misdiagnosis.

## 3. Gender

Alasil et al. [22] recruited a racially diverse sample of healthy subjects, of whom 66% were men. However, these researchers did not find any relationship between gender and RNFL thickness, which corroborated the results of previous studies [23]. However, in recent years, a study conducted in Iran concluded that males tended to have thinner RNFLs than females [24]. A population-based study found that females had a smaller angle between the peak RNFL thickness of the superior-temporal and inferior-temporal sectors [25]. Since gender can affect RNFL thickness, it may be necessary to record the gender of every subject.

## 4. Ethnicity

RNFL variations among different ethnic groups have attracted attention from many investigators. Different ethnicities have different RNFL thicknesses [22, 26]; for example, a study determined that people of European descent were likely to have thinner RNFL than people of other ethnicities [27]. Similarly, other studies found that normal Caucasian subjects had thinner RNFLs than normal subjects of Hispanic or Asian heritage [13, 22]. When African Americans were measured, their average RNFL thickness was 101.1 mm [13], which was thicker than the 98.1 mm reported in Caucasian subjects [13, 28]. A Singaporean study reported thinner RNFLs in the local population than in Chinese people [29].

Some of the above studies used similar OCT scanning parameters but produced different results. This might be because the database used by each manufacturer of OCT equipment varies regarding the number of samples, the eligibility criteria, and ethnic makeup [30]. This highlights the importance of taking ethnic differences into account when ocular phenotypes such as disc size, RNFL thickness, and cup volume are measured.

## 5. Spherical Equivalent (SE)

The search for an association between spherical equivalent and retinal nerve fibre layer thickness has spanned more than twenty decades. Myopia is divided into two subtypes: axial myopia and refractive myopia. The latter is caused mainly by the change in curvature of the cornea or lens. Numerous studies have found that RNFL thickness is negatively associated with the degree of myopia [31]. Sectoral reductions in the average and nontemporal RNFL thickness have been reported [32], while the temporal thickness was found to be significantly increased [33, 34]. This thickening may be attributed to the convergence of the superotemporal and inferotemporal RNFL bundles. Compared to choroid thickness, global peripapillary RNFL thickness is an independent predictive factor of best-corrected visual acuity (BCVA) [35]. Nontemporal sectors, especially the upper and lower parts of the disc, become thinner in adults and children, as well as in the case of ultrahigh myopia [36]. However, Chen et al. [37] reported that there was no relationship between the global RNFL thickness and SE in a teenage group. It can be postulated that RNFL thickness may not change with the degree of myopia at a young age. A study of healthy myopic subjects with more than 5 years of follow-up found that refractive error affected the overall RNFL thickness with a thinning rate of 0.208–0.305 *μ*m/yr [20]. Other cross-sectional studies that included different age groups found slower thinning rates than longitudinal studies [13, 38, 39]. The inferior quadrant has the fastest thinning rate (−0.778 *μ*m/yr), while the superior quadrant is slightly slower (−0.524 *μ*m/yr) [20]. It was demonstrated that the lamina cribrosa in the superior and inferior quadrants was weaker, making them likely to sustain glaucomatous damage [40]. Although there were differences between the above studies, longitudinal studies can better reflect true changes in the RNFL thinning rate in the same participant.

Anisometropia is defined as the difference in interocular refraction reaching more than 1 D [41]. Even in the same people who had myopic anisometropia, the mean RNFL thickness of the eyes that were more myopic was found to be significantly thinner than that of the fellow eyes [42].

Topical atropine is widely used to paralyze the ciliary muscle to identify whether patients have true myopia. In adults, it can also be used to alleviate pain in uveitis. Theoretically, tropical atropine may increase intraocular pressure and then damage the RNFL. Additionally, atropine, as a nonselective muscarinic antagonist, can cause vasoconstriction, leading to hypoperfusion of the optic nerve. However, a study followed myopic children aged 5–15 years for at least 1 year, who used 0.25% atropine to prevent myopic progression, and found that atropine use did not significantly affect optic nerve-related parameters such as peripapillary RNFL thickness in children [43]. In adults, whether RNFL thickness changes after long use of tropical atropine needs further investigation.

The relationship between SE and RNFL achieved a consensus among most studies. These studies, however, included mostly adults. In children, it remains uncertain whether such an association exists; larger samples are needed to identify a clear answer.

## 6. Axial Length (AL)

AL is recognized as the primary factor in myopia. However, it is challenging to distinguish early glaucomatous damage in myopic eyes when some ocular morphological abnormalities are associated with eye elongation.

Among healthy emmetropic eyes, all quadrants of the RNFL except the temporal sector decreased with increasing AL [44, 45]. The global RNFL thickness decreased by 3.086 *μ*m with each additional millimetre of AL, and the decrease in the inferior quadrant of the RNFL showed the strongest relationship with longer AL (4.46 *μ*m/mm) [19]. Elongation and thinning of the sclera and the retina, which spread the nerve fibres over a larger surface area, could cause thinning of the RNFL in people with myopia. There was still no anatomical evidence showing that the elongation of the AL could cause retinal ganglion axon degeneration. Another potential mechanism was postulated to involve less stimulation of ganglion cells due to negative lenses in myopia, as demonstrated by a study on chickens [46]. Myelination depends on activity and is highly plastic. If ganglion cells are subjected to low stimulation levels, their axons might traffic less information, causing axon demyelination and thinning. However, an experimental study on goldfish found that this demyelination could be reversed [47]. The temporal quadrant is where papillomacular bundles exist and macular fibres are located mainly near the upper and lower horizontal sutures towards the optic disc. The thicker temporal RNFL has been attributed to RNFL redistribution as the result of eye elongation that drags the retina towards the temporal horizon [34, 48]. Normally, the fovea is located vertically below the optic nerve head (ONH) in emmetropic eyes. The change in AL will change the position of the fovea related to the ONH, which will cause vertical asymmetry of the RNFL distribution as the fovea shifts into the inferior part of the eye [49]. A population-based study found that the angle between the peaks of peripapillary RNFL thickness in the upper and lower hemispheres decreased with increasing AL by −5.86°/mm [25]. However, this change is not always associated with AL. For children, there was no relationship between them [50, 51]. The mean RNFL thickness in 7-year-old Chinese children was similar to that in 12-year-old Chinese children, which was 102.01 ± 8.02 *μ*m and 103.08 ± 9.01 *μ*m, respectively [18]. Therefore, we can speculate that RNFL changes along with AL may not occur at an early stage during myopia progression. OCT measurements of the RNFL can be influenced by the AL-induced magnification effect. The scan circle is projected to be larger on the retina than the actual circle after elongation of the eyeball [52]. Theoretically, the RNFL thickness around the optic disc becomes thinner as the distance to the optic disc increases. Most studies did not adjust this magnification. As demonstrated previously, after adjusting magnification, global and nontemporal RNFL measurements were negatively associated with AL [53], while global RNFL thickness became thicker in axial myopia [54]. Due to the considerable impact of the ocular magnification effect on the peripapillary RNFL measurements, routine correction for this factor using Littmann's formula, described by Bennett et al. [55], should be considered.

It is widely known that average RNFL thickness in the peripapillary region and the nontemporal quadrants are negatively correlated with AL, but this relationship does not always exist, especially in high myopia when a posterior staphyloma is located temporally to the fovea. Posterior staphyloma usually appears after extreme elongation of the eye, and AL might be measured differently. In this case, ultrasound resonance is recommended to help locate the posterior eye wall accurately. Therefore, in axial myopia, the main nasal quadrant changes along with the AL. When the temporal RNFL thins in axial myopia, glaucoma should be considered.

## 7. Corneal Astigmatism (CA)

It has been reported that cylindrical refractive error can also influence measurements of the RNFL by OCT by distorting retinal images. The retinal image became elliptical in cylindrical refractive error, and its shape varied according to the axis of astigmatism [56]. Previous studies reported that RNFL thickness decreased in the temporal quadrant due to the greater distance between the fovea and the superotemporal and inferotemporal peaks than in the normal CA group. However, global average and nontemporal RNFL thickness did not show significant differences [57].

How astigmatism affects RNFL thickness and optic disc parameters remains uncertain. This might be due to CA-induced ocular magnification among high myopia, which might result in an increased distance from the disc rim border to the disk front surface and then reflectivity detected by OCT changes.

## 8. Characteristics of the Optic Disc

### 8.1. Optic Disc Area

As the convergence of the retinal nerve fibre layer constitutes a part of the optic disc, its thickness may be associated with the disc area. As shown in a previous histomorphometric study [58], the larger optic disc accommodated more retinal nerve fibre axons. Clinical research also concluded that the optic disc area had a positive relationship with AL [48, 59–61], as long eyes with more retinal surfaces could contain more RNFL than normal eyes. In children, a large cup/disc ratio with a large disc area is usually recognized as a normal sign. However, 13% of them developed glaucoma for just 3 years [62]. RNFL thickness in myopic children with larger cup disc ratios did not change significantly [63]. RNFL losses usually occurred after the age of 50 years, as claimed by Parikh et al. [38]. The relationship between RNFL thickness and large disc size might be attributed to overestimation caused by the shorter distance from the scanning circle to the disc margin, as RNFL thickness becomes thinner with increasing distance from the disc margin [33].

### 8.2. Optic Tilt

Optic tilt means that the long axis of the optic disc forms an angle with the vertical axis [64]. This might be due to scleral stretching during myopia progression [65]. Thirty-seven percent of the myopic population had this anatomic feature [66]. Since it is common in myopic eyes, changes in RNFL thickness distribution should be considered.

In myopic eyes, the appearance of optic disc tilt could indicate the development of glaucomatous optic discs [65]. Therefore, when eyes show obvious tilt of the optic disc, attention should be given to the possibility of glaucoma. Eyes with optic tilt tend to have thicker temporal RNFL thickness due to retina convergence towards the macular region [67]. For quadrants, the RNFL thickness adjacent to the elevated disc rim became thinner [68]. The preferential location of RNFL thinning might be attributed to the lamina cribrosa depth profile based on the tilted optic disc axis [69]. In highly myopic eyes, only the thickness of the RNFL at the temporal side of the macula was significantly thickened in the group with tilted optic discs, in both the inner and the outer layers [70]. However, there was no significant difference in overall RNFL thickness between the optic disc tilt group and the nontilt group in healthy eyes [65], which might be due to uneven changes in RNFL distribution associated with optic tilt. Various changes in different areas around tilted optic discs should be considered during RNFL measurements.

### 8.3. Optic Rotation

The optic disc is usually characterized in terms of size and shape [71]. Optic disc rotation can change with the shape of the front part of the eye, such as the cornea. As corneal astigmatism is associated with RNFL thickness, optic rotation may have the same impact on RNFL thickness.

The optic disc can rotate in two ways. When it rotated around the vertical axis, the temporal disc border usually moved backward, while the corresponding nasal disc margin moved forward. When rotating along the horizontal axis, the inferior disc border moves backward, and the superior disc border moves forward.

In high axial myopia, the optic disc rotates more horizontally, causing thinner superior nasal RNFL thickness and thicker inferior nasal RNFL thickness due to the position of the fovea being more horizontal to the optic disc [72]. This location change will cause uneven distribution of the RNFL.

## 9. Retinal Vasculature

Histological studies have shown that vascular networks exist in the RNFL, ganglion cell layer, inner plexiform layer, inner nuclear layer, and outer plexiform layer [73–75]. The vasculature supplies neuroretinal layers with oxygen and nutrients. Therefore, they would have an impact on RNFL thickness.

Wang et al. [76] demonstrated that in high myopia, the peripapillary flow index and vessel density in the retina were decreased using OCTA. Narrowed retinal arterioles have also been reported in myopia [77]. OCTA, a novel ocular blood imaging technology, can image and quantify retinal microcirculation with good sensitivity and reproducibility. The reduction in retinal perfusion caused by AL may also play a role in RNFL thickness alteration [78]. The RNFL may be more affected by the position of retinal vessels when the peaks of peripapillary RNFL profiles change with superior and inferior vascular structures.

Previous studies emphasized large retinal vasculature structures. The larger retinal vessels play a significant role in peripapillary RNFL thickness, since they comprise 13% of the total RNFL thickness [16]. Therefore, the calculation of RNFL thickness should subtract blood vessels. The association between RNFL thickness and blood vessels in myopia can be explained by the reduced metabolic demand. It has been presumed that decreased density of retinal microvasculature is probably associated with the closure or degeneration of capillaries during RNFL loss.

Vascular alterations may provide insight into how the pathophysiological mechanism occurs in high myopia. Thus, some preventive measures and alternative treatments aimed at these vascular alterations may be the goal of future studies. However, it is still challenging to determine whether decreased vascular density occurs before or after ocular structural changes, such as RNFL thinning, according to a cross-sectional study. Future longitudinal studies are needed to help us better understand this.

## 10. Peripapillary Detachment

A peripapillary detachment in pathologic myopia (PDPM) was described as a lesion inferior to the optic disc along the inferior margin of the myopic conus, which is well circumscribed, dome shaped, and yellow orange [79]. It consisted of peripapillary intrachoroidal cavitation (PIC) and peripapillary neurosensory retinal detachment (PNRD). The former was a hyporeflective space below the normal RPE plane, while the latter was between the neural retina and the RPE, with a prevalence of 9.01% and 8.19%, respectively, in high myopia [80]. The pathogenesis of PDPM remains unclear. It has been demonstrated that PDPM is not influenced by AL, SE, sex, or age. No association was observed with visual acuity. In high myopia, patients with PNRD had a thicker average RNFL thickness, while eyes with PIC and those without PDPM showed no significant difference in the average and quadrant RNFL thickness [80]. Since eyes with PNRD did not show any disease, such as papilledema or papillitis, that caused swelling of the RNFL, it was assumed that such eyes had normal RNFL thickness [80]. Therefore, PNRD may cause incorrect measurements of RNFL by OCT. If a clinician ignores the presence of PNRD when performing this examination, the increase in RNFL thickness may be misdiagnosed as pathologic.

## 11. Signal Strength

OCT works based on the ability of light to pass through ocular media. Its ability is qualified as signal quality measured by comparison to a light source from a reference mirror [3]. In previous studies, researchers usually recognized a signal strength above 7 as a credible measurement. Sometimes, it can be affected by many factors, such as changes in the transparency of ocular media, causing poor image quality. Ray et al. [81] reported that ocular pathologies such as cataracts, uveitis, and retinal disease can cause image degradation. Vizzeri et al. [82] also found that signal strength was positively associated with mean RNFL thickness in healthy subjects. When the signal strength decreases for each unit, there will be a decrease of 2 mm in the average RNFL thickness. RNFL thickness measured by OCT increased as patients accepted cataract surgery, which may help by improving signal strength [83]. The internal limiting membrane is usually identified as the upper boundary of the RNFL by algorithms in OCT. Sometimes, a serious opacity in ocular media such as vitreous haemorrhage will be incorrectly identified as the internal limiting membrane, and the RNFL can be measured incorrectly. These segmentation errors can be compensated by the RNFL deviation map. A study was conducted on the change in RNFL thickness with patients who has no light perception vision and concluded that its RNFL thickness remained 30–40 mm because retinal blood vessels and residual glial cells were in it [84]. Therefore, when OCT measurements show 0 mm RNFL thickness, segmentation error should be taken into consideration.

Another scenario can be seen in people wearing soft contact lenses. Some visible deposits mainly composed of protein [85] on the lens surface, similar to cataract and vitreous opacity, can weaken the ability of light from OCT crossing through the eye, causing the reduced signal reflected from the retina [86]. When patients wear contact lenses or long-lasting contact lenses, wearers tend to have thinner RNFL thickness [87]. In addition, corneal swelling induced by contact lens wear could affect the accuracy of RNFL measurements by increasing corneal backscattered light [88]. These might be misperceived as glaucomatous damage.

Signal strength is a reminder of the image quality in OCT. Once signal strength drops below a value of 7 (10 being maximum) in the Cirrus platform, RNFL segmentation will be measured incorrectly. Therefore, clinicians should be careful in measuring RNFL thickness using OCT, especially in those who have ocular opacity and wear soft contact lenses.

## 12. OCT Segmentation Artefacts

Accurate delineation of the retinal layers can guarantee the correct RNFL measurements. It has been reported that at least one segmentation error occurred in 19.9% to 46.3% of scans [89]. Such segmentation artefacts are especially likely to occur in high myopia [90]. This relatively high estimation of the possibility of segmentation errors might be attributed to thinner RNFL thickness caused by elongation of the eye [91]. Therefore, this segmentation error caused by thinner RNFL thickness might result in more errors in both high myopia and glaucomatous eyes since glaucoma is associated with decreased regional RNFL thickness. In addition, when posterior staphyloma exits myopia, retinal layer delineation will be more difficult due to the challenge of identifying the boundary of the eye. In this case, manual correction was especially important. The diagnostic capability of the RNFL thickness in glaucoma measured by OCT significantly improved after manual correction of segmentation errors.

Additionally, when OCT scans are with larger diameters, a segmentation error has more opportunities to occur in larger areas. Scans with larger diameters can measure farther from the optic disc rim. As we mentioned above, the RNFL became thinner with increasing distance to the optic disc. A thinner RNFL is associated with more segmentation errors. Based on this, to increase the diagnostic capability of OCT, some segmentation correction software and a more accurate automated segmentation algorithm are needed.

## 13. Other Confounders

There are still some confounders that have been researched. However, those variables did not show a significant association with RNFL thickness; examples include anterior chamber depth as well as corneal thickness variables. Central corneal thickness was negatively associated with RNFL thickness in myopia [92]. The reason why central corneal thickness affects the RNFL remains unclear. It is known that glaucoma in persons with ocular hypertension is more likely to have a thicker central cornea [93]. Ocular hypertension can indirectly decrease RNFL thickness by influencing retinal vasculature. RNFL thickness changed differently with elevated intraocular pressure (IOP) among peripapillary sectors. The temporal-superior, nasal-inferior, and temporal sectors showed the greatest decline and were thought to be more sensitive to elevated IOP, which could be used to detect glaucomatous damage to RNFL thickness [94]. Suić et al. [95] found that there was a considerable decline in inferior RNFL thickness in people with ocular hypertension. As for the reduction rate, RNFL thickness measured by the Spectralis spectral-domain optical coherence tomography (SD-OCT) declined faster in those who had visual field defect. It lost 1 mm/year faster, which meant a 2.05-times higher risk of developing visual field loss [96]. Therefore, the reduction rate of RNFL thickness can predict glaucoma progression to some extent. However, these results need larger samples to demonstrate.

There have been a few studies on most of these factors. If we want to determine whether they have relationships with RNFL thickness and how they affect, they still need further study.

## 14. Conclusion

RNFL thinning is a nonspecific sign that is present in myopia and glaucoma. Other neurodegenerative diseases, including Parkinson's disease and multiple sclerosis, have also been reported to decrease RNFL thickness. Systematic diseases such as hypertension cause a reduction in RNFL thickness, especially in the superior and inferior quadrants, possibly because of high oxygen demand in these two areas, making it prone to ischaemic damage [97]. Therefore, when people have systematic diseases, the measurement of the RNFL should be considered.

This is a systematic review of the existing research on the determinants of RNFL thickness in myopic eyes. This review shows that in myopia, RNFL thickness is influenced by various factors. This will aid clinicians in accurate measurements of RFNL thickness in myopia and reduce false-positive rates. When detecting abnormal RNFL thickness changes, we should consider comprehensively. To date, most studies have focused on the relationship between SE or AL and RNFL thickness. However, how peripapillary perfusion affects RNFL thickness in myopic eyes needs further research. Additionally, studies on race and gender still need a larger sample to determine their true relationships. With the development of OCTA, it is possible to gain clarity in these areas, making the measurement of RNFL thickness more accurate in myopia. Then, we can detect glaucomatous damage to the retina earlier and prevent the progression of myopia.

## 15. Method of Literature Search

A literature search was performed on the PubMed mainly from 2005 to 2021. Search terms included “high myopia,” “retinal nerve fibre layer,” and their combinations. Further articles were identified from the reference lists of retrieved articles.
